# Factors Associated with the Nutritional Status of Women with Non-Metastatic Breast Cancer in a Brazilian High Complexity Oncology Center

**DOI:** 10.3390/nu15234961

**Published:** 2023-11-29

**Authors:** Roberto Júnio Gomes Silva, Wesley Rocha Grippa, Luiz Claudio Barreto Silva Neto, Oscar Geovanny Enriquez-Martinez, Júlia Anhoque Cavalcanti Marcarini, Raphael Manhães Pessanha, Fabiano Kenji Haraguchi, Luís Carlos Lopes-Júnior

**Affiliations:** 1 Graduate Program in Nutrition and Health, Universidade Federal do Espírito Santo, Vitória 29500-000, ES, Brazilfabiano.haraguchi@ufes.br (F.K.H.); 2 Graduate Program in Public Health, Universidade Federal do Espírito Santo, Vitória 29500-000, ES, Brazil; wgripa@gmail.com (W.R.G.); rafa.09pessanha@gmail.com (R.M.P.)

**Keywords:** breast neoplasms, nutritional status, food, diet, food, and nutrition, risk factors

## Abstract

Background: Breast cancer poses a significant public health concern owing to its high prevalence and the risk of mortality associated with delayed diagnosis and treatment. The aim of this study was to assess the nutritional status of women with non-metastatic breast cancer and to identify factors associated with it. Methods: A cross-sectional observational study was conducted at a High Complexity Oncology Assistance Center in the southeast region of Brazil, with the aim of assessing the nutritional status in women undergoing treatment for stage I, II, or III breast cancer. Patients in palliative care or undergoing reconstructive surgery were excluded. Data collection took place between June 2022 and March 2023 and included questionnaires, physical examinations, laboratory tests, and anthropometric assessments. Nutritional status was assessed using measures such as BMI and skinfold thickness, while nutritional risk was assessed using the Nutritional Risk Screening (NRS-2002) tool. Results: Significant associations were found between nutritional risk and educational level (*p* = 0.03) and BMI (*p* = 0.01). Binary logistic regression analysis revealed a significant association between educational level and nutritional risk, indicating that lower educational level was associated with higher odds of nutritional risk (OR = 4.59; 95% CI = 1.01–21.04; *p* = 0.049). In addition, regarding BMI, it was observed that a BMI above 20.5 kg/m^2^ was associated with a higher likelihood of nutritional risk (OR = 0.09; 95% CI = 0.01–0.89; *p* = 0.039). Conclusions: It is crucial to consider the nutritional status of breast cancer patients, alongside clinical factors, to offer comprehensive and personalized care. Gaining insight into the sociodemographic variables linked to nutritional risk can significantly contribute to our understanding of breast cancer. This knowledge, in turn, can aid in identifying effective strategies for public policy, health promotion, and prevention efforts aimed at tackling this condition.

## 1. Introduction

The World Health Organization predicts that by 2030, cancer will reach approximately 27 million new cases worldwide, with 17 million deaths caused by the disease and around 75 million survivors. The impact will be greatest in low-income countries [1]. The International Agency for Research on Cancer’s (IARC) most recent report on cancer incidence and mortality estimates, GLOBOCAN 2020, projected 19.3 million new cancer cases and 10 million deaths from the disease in 2020, based on geographic variability in 185 countries worldwide [2]. Breast cancer is the most diagnosed malignant neoplasm, accounting for 11.7% of all cases in women, and is the second leading cause of cancer-related death worldwide [2,3].

In Brazil, according to the latest National Cancer Institute’s (INCA) estimate, 704,000 new cancer cases are expected each year during the 2023–2025 triennium. Female breast cancer is the most common cancer in all Brazilian regions (10.5%), excluding non-melanoma skin tumors. Specifically, in women, an estimated 74,000 new cases of breast cancer are projected per year during the 2023–2025 triennium [4].

Breast cancer is a multifactorial disease. Its causes include factors such as age, ethnicity, geographic location, family history of cancer before the age of forty, lack of pregnancy, first pregnancy and childbirth at an advanced age, early menarche, late menopause, exposure to radiation, use of oral contraceptives, and hormonal changes such as excessive estrogen exposure. Lifestyle factors such as alcohol consumption, poor diet, tobacco use, and lack of physical activity also play a role. In addition, a higher body mass index (BMI) is associated with an increased risk of developing breast cancer and a worse prognosis [5,6,7,8].

Alterations in nutritional status in cancer patients are associated with the location, stage, and symptomatology of the disease, as well as the type of antitumor treatment used [9,10]. Furthermore, altered nutritional status leads to changes in body composition due to skeletal muscle loss and reduction of adipose tissue reserves, resulting in variations in body weight [9,11,12].

According to the guidelines established by the European Society for Clinical Nutrition (ESPEN) with the publication of clinical nutrition guidelines in cancer, a comprehensive assessment of nutritional status should be performed shortly after diagnosis [13]. Continuous monitoring is recommended, followed with the implementation of nutritional interventions regardless of the cancer stage [12,13]. The European Society for Medical Oncology (ESMO) suggests the following methods for nutritional assessment: body composition, BMI, dietary intake, C-reactive protein, albumin, systemic inflammation, the Nutritional Risk Screening-2002 (NRS-2002), and the Malnutrition Universal Screening Tool (MUST) [11,13].

The nutritional status of breast cancer patients might be negatively affected by the therapeutic process due to side effects such as mucositis, nausea, and vomiting, which occur together as a cluster of gastrointestinal symptoms, along with anorexia, all of which are determinants of patients’ dietary habits and consequently impact on their nutritional status [14,15,16]. Thus, during antineoplastic treatment, food intake may be impaired and contribute to the deterioration of the patient’s nutritional status.

Specialized nutrition therapy is of paramount importance to help restore and/or maintain the patient’s nutritional status, quality of life, and functionality. However, studies that include factors associated with the nutritional status of stage I, II, and III breast cancer patients, especially those who are hospitalized, remain scarce in the literature. The aim of this study was to assess the nutritional status of women with non-metastatic breast cancer and to identify factors associated with it.

## 2. Materials and Methods

### 2.1. Study Design

An observational cross-sectional study was conducted at a cancer treatment referral hospital (Santa Rita de Cássia Hospital—HSRC) in the southeast region of Brazil.

### 2.2. Ethical Aspects

Ethical approval was obtained from the Research Ethics Committee of the Health Sciences Center (CEP-CCS-UFES) of the Federal University of Espírito Santo, Brazil, with a Certificate of Ethical Appreciation (CAAE) number: 57491022.1.0000.5060 and approval number: 5.400.652. After identifying eligible patients with stage I, II or III breast cancer at the hospital, informed consent was obtained from each patient who voluntarily agreed to participate in this research, and they signed the informed consent form.

### 2.3. Study Sample

The eligibility criteria for this study were as follows: females, over 18 years of age, with an anatomopathological diagnosis of stage I, II, or III breast cancer according to the International Classification of Diseases (ICD-10): C50 (malignant breast neoplasm), and in any phase of antineoplastic treatment. Patients in palliative care, those admitted for reconstructive surgery or non-cancer clinical problems, and/or those with any cognitive impairment that would interfere with their ability to respond to the data collection instruments were excluded from this study.

The sample size calculation was based on the latest estimate of the INCA for the triennium 2023–2025, which projected 74,000 cases of female breast cancer in Brazil, with 84.46/100,000 cases expected for the southeast region [4]. The caseload in the Inpatient Sector A of Santa Rita Hospital (where the data were collected) was 225 cases of breast cancer admitted for different stages of antineoplastic treatment in 2019, and 182 cases in 2020, resulting in an average of 204 cases per year for the biennium. We considered only the prevalence of breast cancer cases in 2019 in our calculation to reduce the bias caused by the COVID-19 pandemic, which significantly affected patient flow and hospitalizations in 2020. Using the formula for sample size calculation [17]—n = N · Z^2^ · p · (1 − p)/Z^2^ · p · (1 − p) + e^2^ · N − 1 where n is the calculated sample size, N is the population, Z is the standard normal variable, p is the true probability of the event, and e is the sampling error—and considering the population of breast cancer patients diagnosed at Santa Rita Hospital in 2019 (*n* = 225), based on the ICD-10: C50 (malignant breast neoplasm), setting α at 5% (sampling error), with a confidence level of 95%, β at 0.2 (giving 80% power of the test), and with a minimum percentage of 12%—the sample size for this research was determined to be 95 patients.

### 2.4. Measures and Data Collection

Data collection took place over 10 months, between June 2022 and March 2023. A sociodemographic and clinical questionnaire was developed by the principal investigators of this study, divided into three components: (I) Medical history (oncological history, past medical history, cardiovascular risks, family history, lifestyle habits, diet, and physical activity). (II) General and specific physical examination. (III) Laboratory tests that were part of the hospital routine and accessed from the patients’ medical records (complete blood count and capillary glycemia).

Nutritional status was assessed using anthropometric evaluation, including weight (kg), height (cm), triceps skinfold thickness (mm), arm circumference (cm), and calf circumference (cm), with each measurement performed three times and the arithmetic mean obtained. Subsequently, the BMI was classified using the World Health Organization guidelines (1995) [18]. The adequacy of triceps skinfold thickness was calculated using the formula developed by Blacknurn and Thornton (1979) [19]. The corrected arm muscle area (CAMA) was determined according to Frisancho (1990) [20], and calf circumference was also measured [21].

In addition, the NRS-2002 instrument was used to assess nutritional risk in the patients. The NRS-2002 is a tool developed by Kondrup et al. (2003) [22] and certified by ESPEN [13]. The first step consists of four questions related to BMI < 20.5 kg/m^2^, weight loss in the last three months, reduced food intake in the last week, and presence of severe illness. In the second step, each criterion was quantified based on nutritional status and severity of illness, and one point was added to the score for patients aged ≥ 70 years. A total score < 3 was classified as “no nutritional risk” and a total score ≥ 3 was classified as “nutritional risk” [22].

### 2.5. Statistical Analysis

Categorical variables were presented as absolute and relative frequencies, and numerical variables were described using measures of central tendency and dispersion. Associations between categorical variables were examined using Pearson’s chi-squared test or Fisher’s exact test when appropriate [23]. Binary logistic regression analysis was used to assess the predictive power of variables, with results presented as odds ratios with 95% confidence intervals (CI) [24]. All analyses were performed using the R statistical software (version 4.2.2) and RStudio software (version 2023.03.1), with alpha set at 5%.

## 3. Results

### 3.1. Participants

Table 1 shows the sociodemographic, lifestyle, and clinical variables of hospitalized women with non-metastatic breast cancer. The mean age was 59.1 years, with the majority falling within the age range of 50–64 years (53%). Self-reported ethnicity was predominantly mixed (50%), and 70% had a monthly income of one minimum wage. In terms of education, 44% had completed primary school. In terms of occupation, 43% identified themselves as homemakers. Most women were married (50%) and had two or more children (59%). Regarding lifestyle variables, most women did not use tobacco (93%) or consume alcoholic beverages (86%).

Regarding the breast cancer characteristics, three histologic types were observed, with ductal in situ (38%) and invasive carcinoma (33%) being the most common. In terms of the TNM stage, the most common categories were T2N2M0 (29%) and T3N1M0 (21%). In terms of staging, most patients were classified as stage I (51%), followed by stage III (36%). Regarding comorbidities, most patients did not have systemic arterial hypertension (71%) or diabetes mellitus (87%), or dyslipidemia (96%). Regarding surgical procedures, most patients had undergone previous surgery (64%). Finally, regarding glycemic levels, 40% of patients had normal levels, 27% were classified as pre-diabetic, and 33% had diabetes.

### 3.2. Anthropometric Assessment

Table 2 shows the anthropometric characteristics of hospitalized women with non-metastatic breast cancer. Mean values for weight, height, and BMI were observed, with 77% of these women classified as overweight. It was observed that 76% of the subjects were at increased or very increased risk. Regarding the mean triceps skinfold thickness, 40% of the subjects were classified as overweight. The mean of corrected arm muscle area status has indicated that the majority (58%) of participants were classified as eutrophic. The classification of mean arm muscle circumference (AMC) measurement indicated that 75% of the subjects had adequate muscle mass. Finally, the calf circumference measurement yielded a mean of 33.72 ± 3.74 cm, indicating that 74% of the individuals were classified within the range considered eutrophic, while 26% were classified as undernourished.

### 3.3. Nutritional Status

Table 3 shows the Nutritional Risk Screening assessment. It was found that 25% of the sample had some level of nutritional risk, with 93% of patients not having a BMI below 20.5 kg/m^2^ and 64% not having experienced weight loss in the last 3 months. Regarding reduced food intake, no patient reported this condition. Regarding severity of illness, no patient was classified as severely ill.

Regarding nutritional status according to the NRS tool, 62% of the patients were classified as normal or eutrophic, and there were no cases of severe malnutrition. In terms of severity of illness, 59% of the patients were classified as mildly ill. The total NRS score indicated that only 25% of women were at nutritional risk.

### 3.4. Factors Associated with Nutritional Status

Table 4 shows the results of the Nutrition Assessment in the sample when considering sociodemographic and clinical characteristics. No significant associations were found between the nutritional scale and age, ethnicity, income, number of children, or marital status. However, statistically significant associations were observed between the NRS scores and patients’ level of education, suggesting that lower levels of education may be associated with higher nutritional risk. In addition, significant associations were found between the NRS dietary score and BMI. These associations underscore the importance of considering multiple clinical and social factors when assessing an individual’s nutritional status.

### 3.5. Binary Logistic Regression Model

Table 5 presents the binary logistic regression analysis, which showed a significant association between educational level and nutritional risk, indicating that lower educational level is associated with a higher likelihood of nutritional risk. However, regarding BMI, it was observed that a BMI above 20.5 kg/m^2^ was associated with a higher likelihood of nutritional risk.

## 4. Discussion

In this study, we examined a diverse cohort of participants characterized with a wide age range, predominantly low-income individuals, primarily housewives who were married, and individuals who did not use alcohol and tobacco. Furthermore, the participants in this study reported no comorbidities, but a high prevalence of overweight individuals was identified. Upon analyzing the association between nutritional risk and various factors using the NRS nutritional score, we discovered a significant association with both schooling levels and BMI.

The analysis of sociodemographic variables provides crucial insights for understanding and guiding the diagnosis and care of these participants. We observed an average age of 59.1 years, aligning with the incidence of cancer in women over 50 years. This finding underscores the significance of the age range considered in analyzing and planning targeted interventions for this specific group of women [25]. The participants in this age range are going through menopause, characterized with hormonal changes that lead to estrogen production in peripheral tissues, especially in adipose tissues. This condition can result in fat accumulation [26,27]. These findings can help explain the high BMI observed in this sample.

Our analysis revealed that a significant portion of the participants (corresponding to 77%) were overweight. These results are consistent with the existing literature, which recognizes being overweight as an established risk factor for the development of cancer [28]. The association between overweight individuals and cancer is well documented and covers various types of malignancies, such as cancers of the breast [29], colon [30], kidney [31], and prostate [32]. Therefore, these findings reinforce the importance of preventive measures and interventions for weight control as an integral part of public health efforts aimed at reducing cancer risk.

The relationship between cancer and obesity remains a complex subject, prompting extensive research. To gain a deeper understanding of this association [33], one hypothesis proposes that an elevated level of estrogen may be linked to the development of breast cancer, particularly in postmenopausal women [34]. Another possible hypothesis suggests that obesity contributes to the increased circulating insulin and insulin-like growth factor 1 (IGF-1), which, in turn, can promote cell proliferation and tumor growth [35]. Numerous epidemiological studies support the correlation between overweight and obese individuals and an elevated risk of cancer [36,37].

Apart from being a well-known risk factor for cancer development, obesity poses a significant public health concern. Data from Vigitel (2021) [38] indicate that approximately 51.5% of the population in Vitória and around 57.5% of the Brazilian population are overweight, presenting alarming statistics that underscore the urgent need for effective measures to address obesity and its consequences. These implications become even more worrisome when considering the estimates provided by INCA for the triennium 2022–2025, which project approximately 244 thousand cases of cancer in Brazilian women during this period, with nearly 50% of these cases concentrated in the southeast region of the country [4].

The municipality where this study was conducted mirrors the national landscape concerning overweight and obese individuals. An ecological study, utilizing data from the Food and Nutrition Surveillance System of Brazil (SISVAN), assessed the prevalence of these health issues from 2009 to 2018. The results demonstrated a consistent upward trend of overweight and obese individuals across all age groups, with a particular emphasis on children and adult women in the south and central region of the state of Espírito Santo. These findings are in line with our study’s results [39], further supporting the evidence of this concerning health trend.

The nutritional assessment using the NRS scale revealed that most of the sample (approximately 75%) presented low nutritional risk. These findings are consistent with other studies that have also reported favorable nutritional conditions in most evaluated cancer patients [40,41]. However, it is crucial to emphasize that a notable percentage of patients in this study are in the early stages of cancer, where the process of nutritional depletion has not yet commenced, in contrast to advanced stages.

Regarding the association indicating a higher risk of malnutrition in patients with a BMI above 20.5 kg/m^2^, this result aligns with the existing literature, which has found associations between obesity and more developed stages of breast cancer [42]. Moreover, obesity and being overweight, in breast cancer patients, have been linked to worse survival rates [43,44].

A statistical correlation was identified between the NRS nutritional score and the level of education, suggesting that lower levels of education may be associated with a higher nutritional risk. This association is discussed in other studies that also point to a connection between education levels, cancer diagnosis, and the initiation of treatment. Factors such as lack of information or limited access to health plans and medical consultations have been cited as potential causes of this association [45,46].

It is important to emphasize that the educational level often serves as an indirect indicator of income, which, in turn, is associated with access to healthcare services. Moreover, delays in commencing treatment or diagnosis have been linked to a deterioration in the patient’s health status and survival rate [47]. Numerous studies have shown that socioeconomic factors are associated with a poorer prognosis among cancer patients [48]. Consistent with the existing literature, a multicenter study conducted by Rosa et al. (2020) [49] revealed that patients treated in public hospitals, besides having lower educational attainment, were diagnosed at more advanced and aggressive stages of breast cancer.

In a cross-sectional study that examined the trend of breast cancer incidence in São Paulo, ethnicity was found to play a significant role. Black women exhibited an increasing trend in the occurrence of breast cancer, while white women showed a decreasing trend during the period from 2000 to 2017. These results suggest potential factors such as delays in diagnosis, challenges in accessing high-complexity services, or biological differences between racial groups [50]. It is important to note that although our study did not demonstrate a direct relationship between race and cancer, there is a notable observation that more black and mixed color women are at risk of malnutrition compared to white women.

When analyzing the relationship between schooling and breast cancer, various studies consistently reveal that higher education levels are associated with lower mortality rates. This connection is attributed to education often being used as an indirect indicator of income [51,52,53], and individuals with higher incomes usually have better access to quality healthcare and improved living conditions.

Moreover, an examination of breast cancer mortality in Brazil indicates an increase in mortality rates among women aged between 50 and 54, with even higher rates observed in older age groups [54]. This pattern emphasizes the critical importance of implementing screening and early detection strategies targeted at younger age groups. Furthermore, it underscores the urgent need for awareness programs aimed at promoting breast health and fostering better access to health services. Additionally, it is important to emphasize that there are various therapeutic approaches available for the treatment of non-metastatic breast cancer. The choice among these methods, such as surgical interventions, radiotherapy, systemic chemotherapy, and hormonal therapy, depends on the specific conditions of each patient. These therapeutic options also have a significant impact on the relationship with the individual’s nutritional status [15,55]. Several studies elucidate such connections. For instance, in Ethiopia, it is reported that the majority of individuals participating in chemotherapy treatment for breast cancer present a nutritional status primarily classified as moderate to severe malnutrition, which translates to a lower survival rate [56]. This type of treatment is characterized with decreased appetite and reduced dietary habits, which is associated with the deterioration of nutritional status, often already compromised in cancer patients. These reports encourage initiatives to identify nutritional status and lifestyle patterns as a focus in cancer care and treatment [57].

In contrast, other research pointed out that the chemotherapy treatment used can cause weight gain in some patients, because the antineoplastic agents used (when associated with the use of glucocorticoids) often influence water retention and increase body fat [15]. These findings were also found by another study [55], where women undergoing chemotherapy had a 65% greater risk of gaining weight when compared to those undergoing radiotherapy or hormone therapy.

It is worth noting that the results observed in the participants of this study do not indicate malnutrition; on the contrary, the majority of them are overweight. This can be attributed to the specific characteristics of the participants, particularly the fact that they are not in advanced stages of cancer, which influences their nutritional status.

### 4.1. Clinical and Research Implications

The clinical and research implications of this investigation are profound, as they firmly establish breast cancer as a multifactorial disease. Often, the focus is solely on disease treatment and diagnosis, while neglecting other associated risk factors that play a pivotal role in its development and progression. This study offers a comprehensive perspective by identifying and envisioning such risk factors. Understanding the complexity and interconnectedness of these factors is essential to improve the care and prevention of this disease.

Moreover, by considering sociodemographic and nutritional variables, this study emphasizes the significance of approaching breast cancer in a holistic manner. This information enables us to direct efforts towards developing more effective public policies aimed at promoting women’s health and breast cancer prevention. By addressing these multifaceted aspects, we can enhance our understanding and approach to combating breast cancer, leading to improved outcomes and a greater impact on women’s well-being. Since the treatment of non-metastatic breast cancer includes various combinations of surgery, radiation therapy, as well as systemic chemotherapy, and hormone therapy, it is recommended that future studies consider treatment-induced nutritional changes in the analyses, for instance, stratifying by each type of antineoplastic treatment.

### 4.2. Strengths and Limitations

This study has some limitations that should be acknowledged. Firstly, its single-centered nature restricts the generalizability of the findings to a broader population. Secondly, the cross-sectional design included patients undergoing various antineoplastic therapy schemes and at different stages, potentially influencing their nutritional status. Additionally, the small sample size, particularly in the group of women with stage III disease, may limit the statistical power and precision of the results. Also, it was not possible to stratify the analyses by the types of antineoplastic treatments received by the participants which may affect nutritional status. Despite these limitations, this study also boasts several strengths. Notably, the inclusion of a sample with the same non-metastatic tumor type ensures a more homogeneous group for analysis. Moreover, this study benefits from the reliability of anthropometric measurements and the use of the NRS scale to assess nutritional status, both of which are methods recommended by esteemed organizations such as ESMO, ESPEN [13], and the Global Leadership Initiative in Malnutrition (GLIM) [58].

## 5. Conclusions

Epidemiologic and dietary changes influence nutritional status, including that of oncology patients. In this study, attention is drawn to the fact that most of the population was classified as overweight, and that factors such as educational level and BMI were associated with this outcome. Therefore, understanding the specific characteristics of this population, as well as the risk factors for this disease, is essential for a better understanding of this pathology and for the formulation of more effective and equitable public policies.

## Figures and Tables

**Table 1:** Sociodemographic and clinical characterization of hospitalized women with non-metastatic breast cancer (*n* = 100).

Variables		*n*	%
Age (years)			
	Mean (Standard Deviation)	59.15 (10.27)	-
Age group			
	<50 years	15	15.00
	50–64 years	53	53.00
	≥65 years	32	32.00
Self-reported ethnicity			
	White	38	38.00
	Black	12	12.00
	Mixed	50	50.00
Income			
	<1 Minimum wage	7	7.00
	1 minimum wage	70	70.00
	2 minimum wages	16	16.00
	≥3 minimum wages	7	7.00
Education			
	No education	9	9.00
	Elementary school	45	45.00
	High school	32	32.00
	Higher	14	14.00
Occupation			
	Homemaker	43	43.00
	Housekeeper	22	22.00
	Other	35	35.00
Marital status			
	Single	21	21.00
	Married	50	50.00
	Widow	15	15.00
	Divorced	14	14.00
Children			
	None	16	16.00
	1	25	25.00
	≥2	59	59.00
Smoking			
	No	93	93.00
	Yes	7	7.00
Alcohol consumption			
	No	86	86.00
	Yes	14	14.00
Histological type			
	Invasive carcinoma	33	33.00
	Ductal in situ	38	38.00
	Lobular in situ	29	29.00
TNM *			
	T1N1M0	8	8.00
	T1N2M0	13	13.00
	T2N1M0	14	14.00
	T2N2M0	29	29.00
	T3N1M0	21	21.00
	T3N2M0	6	6.00
	T4N2M0	5	5.00
	T4N3M0	4	4.00
Cancer stage			
	I	51	51.00
	II	13	13.00
	III	36	36.00
Systemic Arterial Hypertension			
	No	71	71.00
	Yes	29	29.00
Diabetes Mellitus			
	No	87	87.00
	Yes	13	13.00
Dyslipidemia			
	No	96	96.00
	Yes	4	4.00
Previous surgeries			
	No	36	36.00
	Yes	64	64.00
Glycemic status			
	Normal	40	40.00
	Pre-diabetic	27	27.00
	Diabetic	33	33.00

* TNM—Tumor-Node-Metastasis.

**Table 2:** Anthropometric characteristics of hospitalized women with non-metastatic breast cancer (*n* = 100).

Variable		*n*	%
Weight (kg)			
	Mean (Standard Deviation)	71.53 (12.52)	-
	Median	71.00	-
Height (m)			
	Mean (Standard Deviation)	1.61	-
	Median	0.05	-
BMI (kg/m^2^)			
	Mean (Standard Deviation)	27.52 (4.63)	-
	Median	27.06	-
BMI status			
	Malnutrition	2	2.00
	Eutrophy	21	21.00
	Overweight	52	52.00
	Obesity	25	25.00
Waist circumference status			
	Normal	24	24.00
	Increased risk	26	26.00
	High risk	50	50.00
Triceps skinfold thickness status			
	Severe malnutrition	15	15.00
	Moderate malnutrition	8	8.00
	Mild malnutrition	9	9.00
	Eutrophy	28	28.00
	Overweight	9	9.00
	Obesity	31	31.00
Arm circumference status			
	Severe malnutrition	0	0.00
	Moderate malnutrition	7	7.00
	Mild malnutrition	14	14.00
	Eutrophy	58	58.00
	Overweight	21	21.00
	Obesity	0	0.00
Corrected arm muscle area status			
	Muscle mass deficit	22	22.00
	Adequate muscle mass	75	75.00
	Excessive muscle mass	3	3.00
Calf (in cm)			
	Mean (Standard Deviation)	33.72 (3.74)	-
	Median	34.00	-
Calf classification			
	Eutrophy	74	74.00
	Malnutrition	26	26.00

**Table 3:** Nutritional Risk Screening assessment of hospitalized women with non-metastatic breast cancer.

Variable		*n*	%
NRS			
BMI < 20.5 kg/m^2^			
	Yes	7	7.00
	No	93	93.00
Weight loss in 3 months			
	Yes	36	36.00
	No	64	64.00
Intake reduction			
	Yes	0	0.00
	No	100	100.00
Serious illness			
	Yes	0	0.00
	No	100	100.00
Nutritional status			
	Normal	62	62.00
	Mild malnutrition	33	33.00
	Moderate malnutrition	5	5.00
	Severe malnutrition	0	0.00
Disease score or severity			
	Absent	0	0.00
	Mild	59	59.00
	Moderate	41	41.00
	Severe	0	0.00
Total NRS Score			
	No nutritional risk	75	75.00
	Nutritional risk	25	25.00

**Table 4:** Nutritional assessment scale dependent on sociodemographic and clinical characteristics of the sample (*n* = 100).

Variables		NRS Total Score		*p*-Value
		No Nutritional Risk	Nutritional Risk	
Age range (years)				0.986 *
	<50 years	11	4	
	50–64 years	40	13	
	≥65 years	24	8	
Ethnicity				0.090 *
	White	31	7	
	Black	11	1	
	Mixed	33	17	
Income				0.590 **
	<1 Minimum wage	6	0	
	1 minimum wage	52	18	
	2 minimum wages	11	5	
	≥3 minimum wages	6	2	
Children				0.1323 *
	None	14	2	
	1	21	4	
	≥2	40	19	
Education				0.035 *
	No education + incomplete elementary	25	3	
	Complete elementary + incomplete high school	32	14	
	Complete high school + incomplete higher	6	6	
	Complete higher	12	2	
Marital status				0.053 *
	Single	19	3	
	Married	31	18	
	Widow	12	3	
	Divorced	13	1	
Histological type				0.934 *
	Invasive carcinoma	24	9	
	Ductal in situ	29	9	
	Lobular in situ	22	7	
TNM				0.392 **
	T1N1M0	4	4	
	T1N2M0	10	3	
	T2N1M0	10	4	
	T2N2M0	24	5	
	T3N1M0	15	6	
	T3N2M0	6	0	
	T4N2M0	4	1	
	T4N3M0	2	2	
Cancer stage				0.452 *
	I	14	7	
	II	55	15	
	III	6	3	
Systemic Arterial Hypertension				0.797 *
	No	55	17	
	Yes	20	8	
Diabetes Mellitus				0.286 *
	No	68	20	
	Yes	7	5	
Glycemic status				0.989 *
	Normal	30	10	
	Pre-diabetic	20	7	
	Diabetic	25	8	
BMI (kg/m^2^)				0.010 **
	Below 20.5	2	5	
	Above 20.5	73	20	

* Pearson’s Chi-square test for independence; ** Fisher’s exact test; Abbreviations: TNM—Tumor-Node-Metastasis.

**Table 5:** Binary logistic regression.

NRS Global Assessment				
Variable		OR	95% CI	*p*-Value *
Ethnicity				
	White	1		
	Black	0.37	0.037–3.72	0.397
	Mixed	3.01	0.89–10.13	0.075
Education				
	No education + incomplete elementary	1		
	Complete elementary + incomplete high school	4.59	1.01–21.04	0.049
	Complete high school + incomplete higher	13.67	2.13–87.68	0.006
	Higher	5.43	0.52–56.42	0.156
Marital status				
	Single	1		
	Married	3.86	0.88–17.02	0.074
	Widow (female)	1.89	0.26–13.97	0.534
	Divorced	0.49	0.03–7.05	0.599
BMI (kg/m^2^)				
	Below 20.5	1		
	Above 20.5	0.09	0.01–0.89	0.039

* Significant when *p* < 0.05.

## Data Availability

Not applicable.

## References

[1] World Health Organization (2022). Cancer Management.

[2] Sung H., Ferlay J., Siegel R.L., Laversanne M., Soerjomataram I., Jemal A., Bray F. (2021). Global Cancer Statistics 2020: GLOBOCAN Estimates of Incidence and Mortality Worldwide for 36 Cancers in 185 Countries. CA Cancer J. Clin..

[3] Siegel R.L., Miller K.D., Wagle N.S., Jemal A. (2023). Cancer statistics, 2023. CA Cancer J. Clin..

[4] Instituto Nacional de Câncer (INCA) (2022). Estimativa 2023: Incidência de Câncer no Brasil.

[5] Brown N.I., Pekmezi D.W., Oster R.A., Courneya K.S., McAuley E., Ehlers D.K., Phillips S.M., Anton P., Rogers L.Q. (2023). Relationships between Obesity, Exercise Preferences, and Related Social Cognitive Theory Variables among Breast Cancer Survivors. Nutrients.

[6] Abrahão C.A., Bomfim E., Lopes-Júnior L.C., Pereira-da-Silva G. (2019). Complementary Therapies as a Strategy to Reduce Stress and Stimulate Immunity of Women with Breast Cancer. J. Evid.-Based Integr. Med..

[7] Sun Y.S., Zhao Z., Yang Z.N., Xu F., Lu H.J., Zhu Z.Y., Shi W., Jiang J., Yao P.P., Zhu H.P. (2017). Risk Factors and Preventions of Breast Cancer. Int. J. Biol. Sci..

[8] Łukasiewicz S., Czeczelewski M., Forma A., Baj J., Sitarz R., Stanisławek A. (2021). Breast Cancer—Epidemiology, Risk Factors, Classification, Prognostic Markers, and Current Treatment Strategies—An Updated Review. Cancers.

[9] Coronha A.L., Camilo M.E., Ravasco P. (2011). A Importância Da Composição Corporal No Doente Oncológico Qual a Evidência?. Acta Med. Port..

[10] Segura A., Pardo J., Jara C., Zugazabeitia L., Carulla J., de Las Peñas R., García-Cabrera E., Azuara M.L., Casadó J., Gómez-Candela C. (2005). An epidemiological evaluation of the prevalence of malnutrition in Spanish patients with locally advanced or metastatic cancer. Clin. Nutr..

[11] Muscaritoli M., Arends J., Aapro M. (2019). From guidelines to clinical practice: A roadmap for oncologists for nutrition therapy for cancer patients. Ther. Adv. Med. Oncol..

[12] Arends J., Bachmann P., Baracos V., Barthelemy N., Bertz H., Bozzetti F., Fearon K., Hütterer E., Isenring E., Kaasa S. (2017). ESPEN guidelines on nutrition in cancer patients. Clin. Nutr..

[13] Muscaritoli M., Arends J., Bachmann P., Baracos V., Barthelemy N., Bertz H., Bozzetti F., Hütterer E., Isenring E., Kaasa S. (2021). ESPEN practical guideline: Clinical Nutrition in cancer. Clin. Nutr..

[14] Whisenant M., Wong B., Mitchell S.A., Beck S.L., Mooney K. (2019). Symptom Trajectories Are Associated with Co-occurring Symptoms During Chemotherapy for Breast Cancer. J. Pain Symptom Manag..

[15] Casari L., da Silva V.L., Fernandes O.A., Goularte L.M., Fanka D.E., de Oliveira S.S., d’Almeida K.S. (2021). Nutritional Status and Gastrointestinal Symptoms in Oncology Patients Receiving Chemotherapy. Rev. Bras. Cancerol..

[16] Han C.J., Reding K., Cooper B.A., Paul S.M., Conley Y.P., Hammer M., Wright F., Cartwright F., Levine J.D., Miaskowski C. (2019). Symptom Clusters in Patients with Gastrointestinal Cancers Using Different Dimensions of the Symptom Experience. J. Pain Symptom Manag..

[17] Santos G.E. Sample Calculation: Online Calculator. http://www.calculoamostral.vai.la.

[18] World Health Organization (2000). Obesity: Preventing and Managing the Global Epidemic.

[19] Blackburn G.L., Thornton P.A. (1979). Nutritional Assessment of the Hospitalized Patient. Med. Clin. N. Am..

[20] Frisancho A.R. (1990). Anthropometric Standards for the Assessment of Growth and Nutritional Status.

[21] World Health Organization (WHO) (1995). Physical Status: The Use and Interpretation of Anthropometry.

[22] Kondrup J. (2003). Nutritional risk screening (NRS 2002): A new method based on an analysis of controlled clinical trials. Clin. Nutr..

[23] Daniel W.W., Cross C.L. (2018). Biostatistics: A Foundation for Analysis in the Health Sciences.

[24] Hosmer D.W., Lemeshow S., Sturdivant R.X. (2013). Applied Logistic Regression.

[25] Instituto Nacional De Câncer José Alencar Gomes Da Silva(INCA) (2020). Dieta, Nutrição, Atividade Física e Câncer: Umaperspectiva Global: Um Resumo do Terceiro Relatório de Especialistas com uma Perspectiva Brasileira.

[26] Tamimi R.M., Spiegelman D., Smith-Warner S.A., Wang M., Pazaris M., Willett W.C., Eliassen A.H., Hunter D.J. (2016). Population Attributable Risk of Modifiable and Nonmodifiable Breast Cancer Risk Factors in Postmenopausal Breast Cancer. Am. J. Epidemiol..

[27] García-Estévez L., Cortés J., Pérez S., Calvo I., Gallegos I., Moreno-Bueno G. (2021). Obesity and Breast Cancer: A Paradoxical and Controversial Relationship Influenced by Menopausal Status. Front. Oncol..

[28] Lennon H., Sperrin M., Badrick E., Renehan A.G. (2016). The Obesity Paradox in Cancer: A Review. Curr. Oncol. Rep..

[29] Sánchez-Jiménez F., Pérez-Pérez A., De La Cruz-Merino L., Sánchez-Margalet V. (2019). Obesity and Breast Cancer: Role of Leptin. Front. Oncol..

[30] Roslan N.H., Makpol S., Mohd Yusof Y.A. (2019). A Review on Dietary Intervention in Obesity Associated Colon Cancer. Asian Pac. J. Cancer Prev..

[31] Hall J.E., Mouton A.J., Da Silva A.A., Omoto A.C.M., Wang Z., Li X., do Carmo J.M. (2021). Obesity, kidney dysfunction, and inflammation: Interactions in hypertension. Cardiovasc. Res..

[32] Tzenios N., Tazanios M.E., Chahine M. (2022). The impact of body mass index on prostate cancer: An updated systematic review and meta-analysis. Medicine.

[33] Pati S., Irfan W., Jameel A., Ahmed S., Shahid R.K. (2023). Obesity and Cancer: A Current Overview of Epidemiology, Pathogenesis, Outcomes, and Management. Cancers.

[34] Hetemäki N., Savolainen-Peltonen H., Tikkanen M.J., Wang F., Paatela H., Hämäläinen E., Turpeinen U., Haanpää M., Vihma V., Mikkola T.S. (2017). Estrogen Metabolism in Abdominal Subcutaneous and Visceral Adipose Tissue in Postmenopausal Women. J. Clin. Endocrinol. Metab..

[35] Lee H. (2022). Obesity-Associated Cancers: Evidence from Studies in Mouse Models. Cells.

[36] Petrelli F., Cortellini A., Indini A., Tomasello G., Ghidini M., Nigro O., Salati M., Dottorini L., Iaculli A., Varricchio A. (2021). Association of Obesity with Survival Outcomes in Patients with Cancer: A Systematic Review and Meta-analysis. JAMA Netw. Open.

[37] Friedenreich C.M., Ryder-Burbidge C., McNeil J. (2021). Physical activity, obesity and sedentary behavior in cancer etiology: Epidemiologic evidence and biologic mechanisms. Mol. Oncol..

[38] Ministério da Saúde, Secretaria de Vigilância em Saúde, Departamento de Análise em Saúde e Vigilância de Doenças Não Transmissíveis (2021). Vigitel Brasil 2021: Vigilância de Fatores de Risco e Proteção para Doenças Crônicas por Inquérito Telefônico: Estimativas Sobre Frequência e Distribuição Sociodemográfica de Fatores de Risco e Proteção para Doenças Crônicas nas Capitais dos 26 Estados Brasileiros e no Distrito Federal em 2021.

[39] Aprelini C.M.D.O., Reis E.C.D., Enríquez-Martinez O.G., Jesus T.R.D., Molina M.D.C.B. (2021). Tendência da Prevalência do Sobrepeso e Obesidade no Espírito Santo: Estudo Ecológico, 2009–2018. Epidemiol. Serv. Saúde.

[40] Italiano Peixoto M., Fernandes Dourado K., Siqueira de Andrade M.I., Silva T.D., França A.K., Mota de Almeida H.R., Araújo de Vasconcelos A., Santana de Melo L. (2017). Comparação Entre Diferentes Métodos de Triagem Nutricional em Pacientes Oncológicos Ambulatoriais. Nutr. Clin. Diet. Hosp..

[41] Abbott J., Teleni L., McKavanagh D., Watson J., McCarthy A., Isenring E. (2014). A novel, automated nutrition screening system as a predictor of nutritional risk in an oncology day treatment unit (ODTU). Support Care Cancer.

[42] Ayoub N.M., Yaghan R.J., Abdo N.M., Matalka I.I., Akhu-Zaheya L.M., Al-Mohtaseb A.H. (2019). Impact of Obesity on Clinicopathologic Characteristics and Disease Prognosis in Pre- and Postmenopausal Breast Cancer Patients: A Retrospective Institutional Study. J. Obes..

[43] Cho W.K., Choi D.H., Park W., Cha H., Nam S.J., Kim S.W., Lee J.E., Yu J., Im Y.H., Ahn J.S. (2018). Effect of Body Mass Index on Survival in Breast Cancer Patients According to Subtype, Metabolic Syndrome, and Treatment. Clin. Breast Cancer.

[44] Yoo T.-K., Han W., Moon H.-G., Kim J., Lee J.W., Kim M.K., Lee E., Kim J., Noh D.-Y. (2016). Delay of Treatment Initiation Does Not Adversely Affect Survival Outcome in Breast Cancer. Cancer Res. Treat..

[45] Cabral A.L.L.V., Giatti L., Casale C., Cherchiglia M.L. (2019). Vulnerabilidade social e câncer de mama: Diferenciais no intervalo entre o diagnóstico e o tratamento em mulheres de diferentes perfis sociodemográficos. Ciênc. Saúde Coletiva.

[46] Medeiros G., Gomes Chagas Teodózio C., Alves Nogueira Fabro E., Sales De Aguiar S., Henrique Machado Lopes A., Cordeiro De Conte B., da Silva E.V., Coelho L.L.P., Muniz N.F., de Carvalho Schuab S.I.P. (2020). Factors Associated with Delay between Diagnosis and Initiation of Breast Cancer Treatment: A Cohort Study with 204,130 Cases in Brazil. Rev. Bras. Cancerol..

[47] Schrijvers C.T., Mackenbach J.P., Lutz J.M., Quinn M.J., Coleman M.P. (1995). Deprivation and survival from breast cancer. Br. J. Cancer.

[48] Marcelino A.C., Gozzi B., Cardoso-Filho C., Machado H., Zeferino L.C., Vale D.B. (2021). Race disparities in mortality by breast cancer from 2000 to 2017 in São Paulo, Brazil: A population-based retrospective study. BMC Cancer.

[49] Rosa D.D., Bines J., Werutsky G., Barrios C.H., Cronemberger E., Queiroz G.S., de Lima V.C.C., Freitas-Júnior R., Couto J.D., Emerenciano K. (2020). The impact of sociodemographic factors and health insurance coverage in the diagnosis and clinicopathological characteristics of breast cancer in Brazil: AMAZONA III study (GBECAM 0115). Breast Cancer Res. Treat..

[50] Costa L.D.L.N., Sardinha A.H.D.L., Verzaro P.M., Lisbôa L.L.C., Batista R.F.L. (2019). Mortality for Breast Cancer and the Conditions of Human Development in Brazil. Rev. Bras. Cancerol..

[51] Lopes-Júnior L.C., Dell’antonio L.S., Pessanha R.M., Dell’antonio C.S., da Silva M.I., de Souza T.M., Grassi J. (2022). Completeness and Consistency of Epidemiological Variables from Hospital-Based Cancer Registries in a Brazilian State. Int. J. Environ. Res. Public Health.

[52] Grippa W.R., Dell’Antônio L.S., Salaroli L.B., Lopes-Júnior L.C. (2023). Incompleteness trends of epidemiological variables in a Brazilian high complexity cancer registry: An ecological time series study. Medicine.

[53] Dell’Antonio L.S., Leite F.M.C., Dell’Antonio C.S.D.S., Souza C.B., Garbin J.R.T., Santos A.P.B.D., Alves M.F., Lopes-Júnior L.C. (2023). Completeness and quality of information about death from COVID-19 in a Brazilian state: A descriptive population-based register study. Medicine.

[54] Meira K.C., Guimarães R.M. (2015). Análise de efeito idade-período-coorte na mortalidade por câncer de mama no Brasil e regiões. Rev. Panam. Salud Publica.

[55] De Cicco P., Catani M.V., Gasperi V., Sibilano M., Quaglietta M., Savini I. (2019). Nutrition and Breast Cancer: A Literature Review on Prevention, Treatment and Recurrence. Nutrients.

[56] Adam R., Haileselassie W., Solomon N., Desalegn Y., Tigeneh W., Suga Y., Gebremedhin S. (2023). Nutritional status and quality of life among breast Cancer patients undergoing treatment in Addis Ababa, Ethiopia. BMC Women’s Health.

[57] Santa Cecília Silva A.A., Lopes T.D.V.C., Teixeira K.R., Mendes J.A., de Souza Borba M.E., Mota M.C., Waterhouse J., Crispim C.A. (2017). The association between anxiety, hunger, the enjoyment of eating foods and the satiety after food intake in individuals working a night shift compared with after taking a nocturnal sleep: A prospective and observational study. Appetite.

[58] Cederholm T., Jensen G.L., Correia M., Gonzalez M.C., Fukushima R., Higashiguchi T., Baptista G., Barazzoni R., Blaauw R., Coats A.J.S. (2019). GLIM criteria for the diagnosis of malnutrition—A consensus report from the global clinical nutrition community. J. Cachexia Sarcopenia Muscle.

